# Supplementary material for: Neutrophils actively swell to potentiate rapid migration
Source: eLife. 2024 Jul 2;12:RP90551. doi: 10.7554/eLife.90551 (PMC11219036; doi:10.7554/eLife.90551)
Supplement: Supplementary file 1. — (A) Chemoattractant-induced swelling genome-wide CRISPR KO screen hits. Rankings determined using MAGeCK Li et al., 2014. Fold change is the median fold enrichment in the dense bin versus the other two bins of the functional guides. FDR is the false discovery rate of each gene given the distribution of negative control guides in the library. See Methods section for details. (B) Volunteer Demographic Information. Demographic information collected for the volunteer donors according to the Institutional Review Board-approved study protocol at the University of California - San Francisco (Study #21-35147). (C) Guides used to make single gene knockouts in HL-60s. The two highest performing guides from the genome-wide screen were chosen to make single gene knockouts in the HL-60 cell line. See Methods for details. [file elife-90551-supp1.docx]

# Supplementary File 1

**Supplemental File 1A. Chemoattractant-induced swelling genome-wide CRISPR KO screen hits**
Rankings determined using MAGeCK (Li et al 2014). Fold change is the median fold enrichment in the dense bin versus the other two bins of the functional guides. FDR is the false discovery rate of each gene given the distribution of negative control guides in the library. See Methods section for details.

| **Ranking** | **HUGO ID** | **Fold Change** | **FDR** | **# Good sgRNAs** | **Common Name** |
| --- | --- | --- | --- | --- | --- |
| **1** | **FPR1** | **4.4617** | **0.00248** | **3** | **FPR1** |
| 2 | STAT3 | 2.8815 | 0.00248 | 4 |  |
| **3** | **SLC9A1** | **3.5013** | **0.00495** | **4** | **NHE1** |
| **4** | **PIK3CG** | **3.3557** | **0.05569** | **4** | **PI3Kγ** |
| 5 | MARS2 | 23.6178 | 0.06374 | 3 |  |
| 6 | NUP35 | 14.5253 | 0.06374 | 3 |  |
| 7 | SKA1 | 3.3554 | 0.06374 | 4 |  |
| **8** | **CA2** | **2.8157** | **0.09942** | **4** | **CA2** |
| 9 | CANX | 3.966 | 0.09942 | 4 |  |
| 10 | COPS3 | 8.2426 | 0.10015 | 4 |  |
| 11 | RPS2 | 18.7498 | 0.07976 | 3 |  |
| 12 | TPR | 9.6024 | 0.16455 | 4 |  |
| 13 | RFT1 | 26.2401 | 0.16455 | 2 |  |
| 14 | SETD1A | 11.1409 | 0.16455 | 2 |  |
| 15 | TP53TG3C | 11.2083 | 0.16455 | 3 |  |
| 16 | UHRF1 | 4.7194 | 0.16455 | 3 |  |
| 17 | THG1L | 10.4411 | 0.16455 | 3 |  |
| 18 | MRPL20 | 10.8506 | 0.16455 | 4 |  |
| 19 | UTP11L | 9.0882 | 0.16455 | 3 |  |
| 20 | TPI1 | 17.976 | 0.16455 | 3 |  |
| 21 | CT47A6 | 2.8505 | 0.16455 | 3 |  |
| 22 | ESCO2 | 11.9249 | 0.1982 | 4 |  |
| 23 | GRXCR2 | 2.8457 | 0.1982 | 4 |  |
| 24 | PARP12 | 21.181 | 0.1982 | 2 |  |
| 25 | NR1H2 | 39.6651 | 0.20894 | 2 |  |
| 26 | PPP2R2A | 4.4472 | 0.20894 | 4 |  |
| 27 | SLC25A26 | 20.7941 | 0.20894 | 2 |  |
| 28 | GANAB | 2.7762 | 0.20894 | 3 |  |
| 29 | MMGT1 | 22.231 | 0.24088 | 2 |  |
| 30 | TCEB2 | 9.1192 | 0.26382 | 4 |  |
| 31 | MRPL17 | 9.465 | 0.26382 | 3 |  |
| 32 | C1QB | 2.7157 | 0.26382 | 4 |  |
| 33 | TMX3 | 3.0917 | 0.26382 | 4 |  |
| 34 | TOPBP1 | 14.0471 | 0.16455 | 3 |  |
| 35 | AP3M1 | 7.9696 | 0.26528 | 2 |  |
| 36 | MSMB | 4.5751 | 0.26528 | 4 |  |
| 37 | WDR83 | 5.4812 | 0.26528 | 4 |  |
| 38 | MARCH7 | 6.5514 | 0.2937 | 2 |  |
| 39 | RNF111 | 2.9736 | 0.2937 | 4 |  |
| 40 | MOGS | 3.5169 | 0.2937 | 3 |  |
| **100** | **SLC4A2** | **2.582** | **0.54636** | **2** | **AE2** |

**Supplemental File 1B. Volunteer Demographic Information**
Demographic information collected for the volunteer donors according to the Institutional Review Board-approved study protocol at the University of California - San Francisco (Study #21-35147)

| Volunteer | Age | Sex |
| --- | --- | --- |
| 1 | 28 | F |
| 4 | 27 | M |
| 5 | 30 | M |
| 6 | 37 | F |
| 8 | 31 | M |
| 14 | 24 | M |
| 17 | 29 | F |
| 18 | 28 | M |
| 19 | 30 | M |
| 20 | 27 | M |

**Supplemental File 1C. Guides used to make single gene knockouts in HL-60s**
The two highest performing guides from the genome-wide screen were chosen to make single gene knockouts in the HL-60 cell line. See Methods for details.

| HUGO gene name | Takara sgRNA ID | Guide Sequence (5’ -> 3’ ) | Internal ID |
| --- | --- | --- | --- |
| FPR1 | sgFPR1_2 | CTACAGTACCTGGTAAAACG | 11 |
| FPR1 | sgFPR1_3 | CTGACAGCAACGATGGACAT | 12 |
| SLC9A1 | sgSLC9A1_2 | TTTGCCAACTACGAACACGT | 13 |
| SLC9A1 | sgSLC9A1_4 | TGAGGAACAGGTCACACATG | 15 |
| PIK3CG | sgPIK3CG_1 | ACTTAACCCTCTCACAGCAG | 16 |
| PIK3CG | sgPIK3CG_3 | GAGAATACGTCCTCCACATG | 18 |
| CA2 | sgCA2_2 | TATGAGTGTCGATGTCAACA | 19 |
| CA2 | sgCA2_4 | TCACTGGAACACCAAATATG | 20 |
| SLC4A2 | sgSLC4A2_2 | ACCTGCCCCACATACCCACA | 21 |
| SLC4A2 | sgSLC4A2_3 | GAAGACGCAGGACCTGATAG | 22 |
| NegCtrl | Neg_Control_Human_0067 | GGTAGGACCTCACGGCGCGC | 23 |
| NegCtrl | Neg_Control_Human_0084 | GCTGTCGTGTGGAGGCTATG | 24 |
